# Identification of two novel Darier disease-associated mutations in the *ATP2A2* gene

**DOI:** 10.3892/mmr.2015.3605

**Published:** 2015-04-09

**Authors:** LIBAO ZHENG, HUILI JIANG, QIN MEI, BIN CHEN

**Affiliations:** 1 Department of Dermatology, Fuzhou Dermatology Hospital, Fuzhou, Fujian 350025, P.R. China; 2 Department of Dermatology, The First Affiliated Hospital of Nanjing Medical University, Nanjing, Jiangsu 210029, P.R. China

**Keywords:** ATP2A2, Darier disease, mutation

## Abstract

Darier disease (DD) is an autosomal dominant inherited skin disorder, characterized by abnormal keratinization, loss of adhesion between epidermal cells, termed acantholysis, and the development of warty papules and plaques on the central trunk, forehead, scalp and flexures. These symptoms are often exacerbated by heat, sweating, sunburn and stress. Mutations in the *ATP2A2* gene, encoding SERCA2, a calcium pump of the sarco/endoplasmic reticulum, are responsible for the disease. The aim of the present study was to investigate two pedigrees of DD and to examine the genetic mutations. DNA was extracted from peripheral blood, which was obtained from four patients with DD, 10 healthy individuals from the two families and 100 ethnicity-matched control individuals, on which subsequent polymerase chain reaction amplification and direct automated DNA sequencing were performed. The results identified two novel missense mutations, p.R603I and p.G749 V. These mutations were not identified in the remaining ten healthy individuals in the same families or in any of the 100 controls. These mutations may contribute to the expanding database of *ATP2A2* gene mutations in patients with DD.

## Introduction

Darier disease (DD; OMIM 124200), also termed keratosis follicularis and Darier-White disease, is an autosomal dominant inherited skin disorder, characterized by abnormal keratinization and loss of adhesion between epidermal cells, termed acantholysis, and the development of warty papules and plaques in seborrheic regions, including the central trunk, forehead, scalp and flexures (1). It has a prevalence of 1:30,000–100,000 worldwide. The lesions usually present in the second decade, and penetrance is almost complete, although the phenotypic expression is variable (2). Certain patients exhibit pits or keratotic papules on the palms and distinctive nail abnormalities, whereas patients with severe disease are handicapped by widespread malodour keratotic plaques. Neurological and psychiatric abnormalities have also been reported in patients with DD (1). The symptoms are often exacerbated by heat, sweating, sunburn and stress (3,4).

The *ATP2A2* gene encoding SERCA2, a calcium pump of the sarco/endoplasmic reticulum, has been identified as the defective gene in the disease (3). *ATP2A2* is positioned on chromosome 12q23–q24.1 and has 21 exons (5). SERCA pumps are important in Ca^2+^ transport between the cytoplasm and the endoplasmic reticulum (ER), using energy from adenosine triphosphate (ATP) hydrolysis, and is, therefore, important in signal transduction for gene expression and cell differentiation (6,7).

The aim of the present study was to investigate the genetic pedigrees of two families with DD, in order to identify novel mutations.

## Materials and methods

### Patients

**Figure 1:**
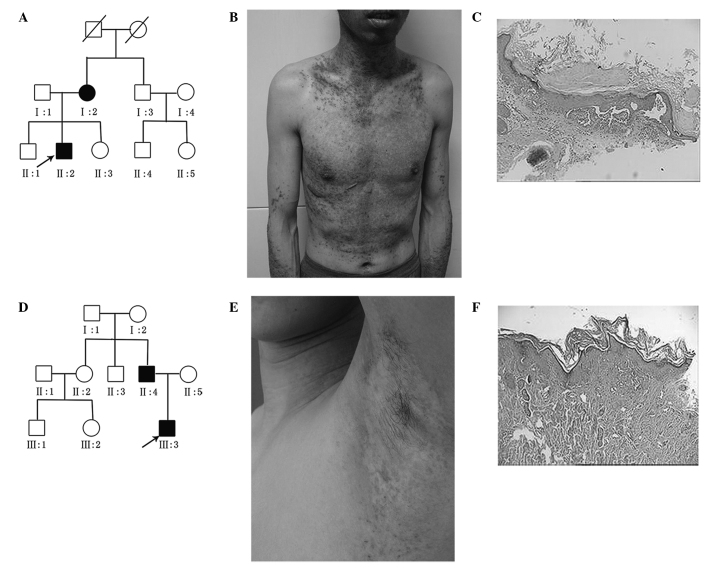
(A) Pedigree of family A, the proband is indicated by the black arrow. (B) Clinical characteristics of the proband of family A, demonstrating keratotic papules in a seborrhoeic distribution. (C) Skin biopsy obtained from family A. The cells were stained using hematoxylin and eosin staining (magnification, x100). (D) Pedigree of family B, the proband is indicated by the black arrow. (E) Clinical characteristics of the proband of family B, demonstrating keratotic papules in a seborrhoeic distribution. (F) Skin biopsy obtained from family B. The cells were stained using hematoxylin and eosin staining (magnification, x100).

In the present study, two families, containing individuals with DD, were recruited. The proband in pedigree A (Fig. 1A), a 36-year-old male, exhibited widespread follicular papules at the age of 26 years (Fig. 1B), and the proband’s mother exhibited similar clinical manifestations. A 0.4 cm × 0.3 cm biopsy was taken from the skin of all patients. The biopsies were fixed in 10% buffered formalin (Shanghai Shiyi Chemicals Reagent Co.,Ltd., Shanghai, China) and routinely processed and embedded in paraffin. Sections (4 *µ*m) were cut and stained with hematoxylin and eosin (Shanghai Shiyi Chemicals Reagent Co.,Ltd.). Histological examination demonstrated hyperkeratosis with parakeratosis, and suprabasal acantholysis with dyskeratotic cells, including corps ronds and grains. The diagnosis of DD was based on clinical and histopathological examinations (Fig.1C). The proband in pedigree B (Fig. 1D), an 18-year-old man, had DD with a 4-year history of papules and hyperkeratotic plaques over the seborrheic area. Physical examination revealed scattered erythema, and follicular papules on the neck and armpit (Fig. 1E). The proband’s father, a 43-year-old male, had been diagnosed with DD for >10 years. This patient manifested greasy, hyperkeratotic papules on the face, forehead, armpit, upper extremities and inguen. None of the patients presented with any neuropsychiatric symptoms. The diagnosis of DD was based on clinical and histopathological examinations (Fig. 1F).

### Genetic analysis

Ethical approval for the present study was obtained from the Ethical Committee of Fuzhou Dermatology Hospital for human studies (Fuzhou, China). Informed consent was obtained from the two family members. A total of 5 ml median cubital vein, basilic vein or cephalic vein blood was collected into a tube with 2% EDTA (0.5 ml), then genomic DNA was extracted from EDTA blood samples, using DNA extraction regents (TIANamp Blood DNA Midi Kit DP332-01, Tiangen, Shanghai, China). Polymerase chain reaction (PCR) was performed in a 25 *µ*l reaction volume, containing 12.5 *µ*l PCR mix, 11 *µ*l double distilled H_2_O, 0.5 *µ*l forward primer, 0.5 *µ*l reverse primer and 0.5 *µ*l (200 ng) genomic DNA. The PCR amplification of *ATP2A2* was performed from the genomic DNA using 19 pairs of primers, spanning all 21 exons and flanking splice sites of the gene, as presented in Table I (5). The amplification was performed at 94°C for 5 min, followed by 32 cycles of 30 sec denaturation at 94°C, 30 sec annealing at 50–62°C, 45 sec extension at 72°C, and termination for 10 min at 72°C (Mastercycler ep gradient S; Eppendorf, Hamburg, Germany).

The purified PCR products were detected by bidirectional direct sequencing using an ABI3130 automated sequencing system (Applied Biosystems, Foster City, CA, USA). DNA, extracted from blood samples obtained from 100 ethnicity-matched healthy individuals, were used as controls. The *ATP2A2* sequence, which was published in the NCBI database (http://www.ncbi.nlm.nih.gov), was used as a reference sequence, which was compared with the experimental results to identify mutations. The nucleotide numbering followed the NM_001681.3 sequence and the amino acid numbering followed the NP_001672.1 *ATP2A2* protein sequence. To determine whether the mutations were pathogenic, *in silico* analysis was performed using PolyPhen-2 (http://genetics.bwh.harvard.edu/pph2/) (8). The categories of pathogenicity included benign, possibly damaging and probably damaging. The mutations were assigned a score between 0 and 1, with the highest score reflecting the closest adherence to be damaging. The mutation (R603I) was predicted to be ‘probably damaging’ with a score of 1 and the mutation (G749V) was predicted to be ‘probably damaging’ with a score of 0.998. Scale-invariant feature transform (SIFT) (http://sift.jcvi.org/) (9), was also used which predicts the effect of a specific amino acid change at the protein level. Amino acids with probabilities <0.05 are predicted to be deleterious. Substitutions at position 603 from R to I and position 749 from G to V are predicted to ‘affect protein function’ with a score of 0.00.

## Results

**Figure 2:**
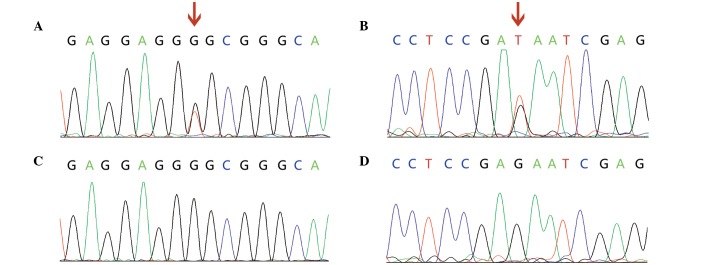
Mutation analysis of the *ATP2A2* gene with Chromas, version 2. 31. (A) Proband of family A exhibited a missense mutation (p.G749V) in exon 15; whereas the (B) proband of family B exhibited a missense mutation (p.R603I) in exon 14. Wild-type alleles of (C) exon 15 and (D) exon 14 of the *ATP2A2* gene are presented.

The entire 21 exons and exon-intron boundaries of the *ATP2A2* gene were sequenced in the two families, in which two novel heterozygous mutations were identified (Fig. 2). In family A, the mutation analysis revealed a nucleotide alteration between G and T (2246G>T) in exon 15 in the two affected individuals, which caused an amino acid substitution from glycine to valine and was designated as p.G749V. In family B, a heterozygous nucleotide change in G1808T in exon 14 was identified in the two affected individuals. This mutation caused an amino acid substitution from arginine to isoleucine acid at position 603 (p.R603I). No *ATP2A2* variants were detected in the unaffected individuals, in either the healthy relatives in the two families or in the 100 ethnicity-matched healthy controls. Furthermore, PolyPhen-2 and SIFT were used to predict the pathogenicity of the mutations. The two novel missense mutations were scored as ‘probably damaging’ using PolyPhen. The prediction scores of the SIFT for each mutation were 0, therefore, p.R603I and p.G749V were considered deleterious mutations, affecting the protein structure and/or function of SCRCA2.

## Discussion

In the present study, two novel mutations in the *ATP2A2* gene were identified following genetic analyses of two families containing patients with DD. The G1808T mutation was detected in codon 603, located within the ATP-binding domain. This missense mutation resulted in the substitution of hydrophilic polar amino acid, arganine, to the hydrophobic amino acid, isoleucine acid, which may have affected the affinity of ATP, leading to alterations in the steric structural and biochemical properties of SERCA2.

The present study also identified G2246T as a second mutation in DD, which was positioned in codon 749. An amino acid substitution from glycine to arginine (G749R) has been previously reported (3), however, the present study demonstrated that a different amino acid substitution at the same amino acid position in the *ATP2A2* gene may have also lead to a steric clash and disrupt the function of SERCA2, leading to DD. These amino acid changes were not observed in the remaining unaffected family members or in the 100 healthy control individuals, therefore, these changes were unlikely to represent silent polymorphisms.

SERCA2, encoded by the *ATP2A2* gene, transports Ca^2+^ between the cytoplasm and the ER and is important in maintaining calcium homeostasis (3,5,10,11). It has been recognized that extracellular calcium ([Ca^2+^]_e_) is important in epidermal differentiation and intra-epidermal cohesion (12). The differentiation of keratinocytes is triggered by increasing the [Ca^2+^]_e_ >0.1 mM (13). Another previous study demonstrated that the calcium concentration in the basal layer in the skin of patients with DD skin is lower than in normal skin *in vivo* (11, 14).

In addition, the keratinocytes of patients with DD exhibit depleted ER Ca^2+^ stores due to the loss of SERCA2 Ca^2+^ transport (15,16). This depletion of Ca^2+^ ER stores potentially triggers the ER stress response, and persistent ER stress, which cannot be overcome due to the defective upregulation of SERCA2, may result in apoptotic keratinocytes, as observed in DD (12). A previous study demonstrated that SERCA2 mutant proteins, which are not degraded by the proteasome, form insoluble aggregates and cause ER stress and apoptosis (17).

Previous studies have also provided evidence to support the role of SERCA2 in the assembly of the desmosomal complex. In normal human keratinocytes, the inhibition of SERCA by thapsigargin, an inhibitor of SERCA2, impairs the trafficking of desmoplakin to the cell surface, and the trafficking of desmoplakin is inhibited in Darier keratinocytes (12). Defective addressing to the desmosomal plaque of desmoplakin, which links the cytoskeleton to the desmosomal complexes, may affect the stability and cell surface expression of desmosomal proteins, impairing cell-to-cell adhesion in the DD epidermis and leading to acantholysis (12,17).

**Figure 3:**
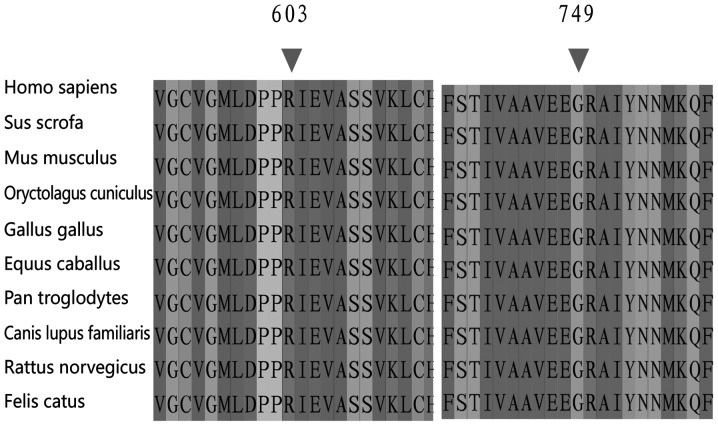
Evolutionary conservation analysis for the two identified *ATP2A2* gene mutations using Clustalx 2.1.

The p.R603I and p.G749V mutations identified in the present study were located within the ATP-binding and stalk domain regions of SERCA2, respectively. These functional domains have been demonstrated to be highly conserved in different species and likely to be critical for the normal function of SERCA2 (Fig. 3) (5). Therefore, it is possible that these missense mutations, which altered polarity and hydrophobicity, may have interfered with the function of SERCA2.

In conclusion, the present study identified two novel missense mutations, p.R603I and p.G749V, in the *ATP2A2* gene in two families containing individuals diagnosed with DD. These results contribute to the expanding database of *ATP2A2* gene mutations. Supplemental functional trials are imperative to confirm the relevance between the mutations and the disease.

## Figures and Tables

**Table I:** Polymerase chain reaction primers for amplification of *ATP2A2* from genomic DNA.

Exon	Forward primer sequence	Reverse primer sequence	Annealing temperature (°C)
1	CGAGGCGGAGGCGAGGAG	GGAGCCGAAGCCCACGCG	62.0
2 + 3	ACCTCCCTCTTGACACATTG	GACAACTCCTAACCACACTG	55.1
4	CGTGCCATTTCTCTTCTAGG	CTCAACACATCAGGAAAAACAG	55.1
5	AGTGTCAGGCAGGTCTTTAC	AGGAAGGGAGGTGCTAAAAC	55.1
6	AGCCTCATTCTCTTCCTTCC	ATGGAGCGAGACTAAAGCAC	55.1
7	CTTGGTGTGGGTCGCAGAG	CCTTTAGAATGATAGCCAGTG	50.3
8	GTTGTATGGCTGGTTGCTTG	GAACAAAGAACCACGACACG	52.0
9	GGTTGTTTGCCTTTGTCCTAA	ATAACAAACACAAATCCCTCTT	50.3
10	GGCGACCATACCCTGCTC	CCCACCCCACCCTTGAAC	55.0
11	TCAGAGGAGGATAAAAATGGC	CTGTAAGTTTGAGGAGATAAGG	52.0
12 + 13	ATTGCCACCCAGTAGTATCC	GAACTGTTTGACCTTTTGCTTG	55.1
14	CTAGAACTTGCCACTTTTATTTA	GAGGCTACTATGTGCTTGTG	50.0
15	TTTCCTCCTGCTTCCCATTC	GCAATCTGGAGAGCAAACTG	55.0
16	TCATTTATTTTTCTGGAGGAGG	CATCTCTGTCTTTTGCTACCC	53.8
17	TGATCTTCGTCCTTGTGGGG	TGATAGATACCGAAACCACAG	53.8
18	GGGTTGGAGCCTGGACTTG	TTTTGGGAAGGGAAGAACTGT	55.0
19 + 20	TCCCCACCTCTCCTTGCTC	CCTCCATCACCAGCCAGTAT	57.5
21	GTTCCTTTTCATCTGTCGCTG	TCTTTTTCCCCAACATCAGTC	53.8
